# Trade-off between Multiple Constraints Enables Simultaneous Formation of Modules and Hubs in Neural Systems

**DOI:** 10.1371/journal.pcbi.1002937

**Published:** 2013-03-07

**Authors:** Yuhan Chen, Shengjun Wang, Claus C. Hilgetag, Changsong Zhou

**Affiliations:** 1 Department of Physics, Hong Kong Baptist University, Kowloon Tong, Hong Kong, China; 2 Centre for Nonlinear Studies, and Beijing-Hong Kong-Singapore Joint Centre for Nonlinear and Complex Systems (Hong Kong), Institute of Computational and Theoretical Studies, Hong Kong Baptist University, Kowloon Tong, Hong Kong, China; 3 Department of Physics, Shaanxi Normal University, Xi'An, Shaanxi Province, China; 4 Department of Computational Neuroscience, University Medical Center Eppendorf, Hamburg, Germany; 5 School of Engineering and Science, Jacobs University Bremen, Bremen, Germany; 6 Department of Health Sciences, Boston University, Boston, Massachusetts, United States of America; 7 Beijing Computational Science Research Center, Beijing, People's Republic of China; 8 Research Centre, HKBU Institute of Research and Continuing Education, Virtual University Park Building, South Area Hi-tech Industrial Park, Shenzhen, China; Indiana University, United States of America

## Abstract

The formation of the complex network architecture of neural systems is subject to multiple structural and functional constraints. Two obvious but apparently contradictory constraints are low wiring cost and high processing efficiency, characterized by short overall wiring length and a small average number of processing steps, respectively. Growing evidence shows that neural networks are results from a trade-off between physical cost and functional value of the topology. However, the relationship between these competing constraints and complex topology is not well understood quantitatively. We explored this relationship systematically by reconstructing two known neural networks, Macaque cortical connectivity and *C. elegans* neuronal connections, from combinatory optimization of wiring cost and processing efficiency constraints, using a control parameter 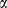, and comparing the reconstructed networks to the real networks. We found that in both neural systems, the reconstructed networks derived from the two constraints can reveal some important relations between the spatial layout of nodes and the topological connectivity, and match several properties of the real networks. The reconstructed and real networks had a similar modular organization in a broad range of 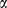, resulting from spatial clustering of network nodes. Hubs emerged due to the competition of the two constraints, and their positions were close to, and partly coincided, with the real hubs in a range of 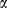 values. The degree of nodes was correlated with the density of nodes in their spatial neighborhood in both reconstructed and real networks. Generally, the rebuilt network matched a significant portion of real links, especially short-distant ones. These findings provide clear evidence to support the hypothesis of trade-off between multiple constraints on brain networks. The two constraints of wiring cost and processing efficiency, however, cannot explain all salient features in the real networks. The discrepancy suggests that there are further relevant factors that are not yet captured here.

## Author Summary

What are essential relationships between fundamental physical constraints and the architecture of neural systems? Most existing investigations have considered a single constraint, either wiring cost or processing path efficiency, and little is known about how characteristic neural network features, such as the simultaneous existence of modules and hubs, are related to the constraints from multiple requirements. Here we emphasized the competition between the global wiring cost and an important functional requirement, path efficiency, as factors in forming Macaque cortical connectivity and *C. elegans* neuronal connections. By comparing real to reconstructed networks using optimization under multiple constraints, we found that several network features are related to the competition of these two constraints, in particular the simultaneous formation of network modules and hubs. However, not all the properties of the real networks could be attributed to these two constraints, suggesting that, likely, there exists additional structural or functional requirements.

## Introduction

It is widely appreciated that complex neuroanatomical networks are the physiological basis for brain dynamics, information processing and mental function [1]–[5]. In the last years, understanding the organization of neural systems and structural-functional relationship using graph theoretical approaches and methods of complex network research has attracted a great deal of interest [6]–[16]. Neural systems possess several pronounced network features, including the small-world property [17]–[19] characterized by short path lengths and high clustering [7], [12], [13], hub nodes with much larger degree than the average node degree of the network [20]–[22] and network modules broadly coinciding with functional subdivisions of the systems [14], [23]–[27]. While these features of neural networks are similar to those in many other real-world complex networks [28]–[30], the mechanisms underlying the formation of such complex network organization are still poorly understood.

The simultaneous existence of modules and hubs is an ubiquitous mesoscopic structural property in neural networks, and may play a significant role in the information processing and functioning of the systems. It was shown that cortical brain connectivity comprises dense communities, which are more densely linked internally than externally [24], [25], [27]. Such a modular organization was observed in structural networks obtained from tract-tracing studies [14], or diffusion spectrum imaging tractography [21], across various species, such as human [21], [31]–[33], cat [34], rhesus Macaque monkey [35] and *C. elegans* [36] as well as in functional networks derived from EEG/MEG, fMRI and MEA experiments [31], [37]–[39]. The modules of the cortical network, in broad agreement with functional subdivisions of the cerebral cortex, are spatially segregated — as the areas within the same functional subsystems (visual, auditory etc.) are mostly spatially co-localized [22], [36], [40], [41]. A recent study [42] showed that that modularity similar to that in human functional networks can be obtained based on an objective function combining the number of common nearest neighbors with a power-law decay of connections over distance, implying that modularity may be closely related to local connections.

Highly connected hub nodes have been shown in the structural network of the human brain, constructed by diffusion tensor approaches and based on 70–90 cortical gray matter areas [43]–[45] or in cortical networks based on 998 region-of-interest [21], as well as in functional networks using fMRI [46] or other imaging techniques (EEG, MEG, MEA) [39], also in other species, such as Macaque cortex [20] and *C. elegans* [47]. Hubs could effectively integrate information that is segregated due to the existence of modules [22]. In agreement with this idea, the identified high-degree hubs were mostly multimodal association regions [16], [22]. Moreover, in a recent study of brain pathology, a MEG study of connectivity provided additional evidence that a degradation of the small-world property in patients with Alzheimer's disease was due to disease-related changes in hubs [48].

What factors influence the formation of modules and hubs is still an open question. Nonetheless, the organization of neural networks is frequently attributed to fundamental constraints, such as metabolic cost [49], signal propagation efficiency [50], evolutionary history [51] and others. It has been speculated that the network organization is the result of an economical trade-off between the physical cost and the functional values of the topology [52]. But it is still not well understood what these functional constraints are and what the relationship is between network properties and functional values, in spite of intensive research on complex brain networks over the last decade.

One of the most extensively discussed aspects is the constraint of wiring cost [53]–[68], which is related to the possible minimization of neural wiring volume [54], [62] or wiring length [55], [58], [59], [61], [63], [65], [69] in the nervous system. Most previous studies investigated whether the actual component placement layout of neural systems has been optimized for wiring minimization, by comparing the actual wiring cost to the perturbed component placement while keeping the network connectivity as in the real systems [55], [59], [63]–[65], [69]. In coarse-grained data sets, it was found that the wiring length of the Macaque prefrontal areas [59] and *C. elegans* ganglia is optimized [55]. However, in other networks, such as those linking Macaque or cat visual cortical areas, the wiring was found not to be fully optimized, but relatively more optimal than other subsets of the cortex [64]. Moreover the wiring of the whole neural network of the Macaque cortex and *C. elegans* neuronal network appeared not to be optimized under the single wiring cost constraint– the total wiring could be decreased to 64% of the original length in Macaque and to 52% in *C. elegans* [63] when applying the component placement optimization (CPO) to minimize the total wiring length while preserving the specific network connectivity. Alternatively, it has been suggested that constraints such as signal propagation efficiency, measured by the global minimization of processing steps across the network, may shape the organization of neural systems [63], [68], [70], [71].

Generally none of the single constraints is likely able to account for all the functional values of the network. However, the processing efficiency perhaps is the most established network measure shown to correlate with functional performance in normal subjects and dysfunction in various brain diseases. As large-scale information processing and communication systems, neural networks favor reducing the number of intermediate transmission steps in order to respond quickly, with the tendency to minimize the average shortest path length (i.e., graph distance) [50], [72]. Minimizing the graph distance has some important functional advantages. First, a small number of intermediate transmission steps might reduce energy consumption during signal processing. In fact, about 50% of the brain's energy is used to drive signal processing, suggested by halved brain energy consumption in deep anesthesia that blocks neural signaling [73]. Second, minimizing the graph distance would increase the speed of signal processing, ultimately leading to faster behavior for decisions and actions [50]. Third, minimizing the graph distance enables neighboring and distant brain areas (or neurons) to receive signals nearly simultaneously to allow synchronous functional processing [74]–[77]. Fourth, since there exists abundant noise in signal transmission (e.g., ionic channel noise or synaptic noise) [50], [78] as well as a high failure rate for signal transmission (between 50% and 90% in individual synapses [50]), minimizing the path length would limit the interference by noise and increase the robustness of neural systems [72]. Indeed, the global efficiency (the inverse of the average shortest paths) of the resting-state brain network has been found to be strongly associated with the intelligence quotient(IQ) [79]–[82]. In disease, it was found that the efficiency of the human cortical network was disrupted in a manner proportional to the extent of white matter lesions [83]–[87]. Therefore, the graph distance/processing efficiency could be taken as a representative measure conferring functional value of brain networks.

To date, however, most studies either considered the influence of just a single constraint (mostly wiring minimization) [53]–[55], [57]–[62], [64]–[66], or evaluated two constraints (such as the metabolic cost constraint and the propagation efficiency constraint) separately [63]. However, this approach does not mean that the two constraints are independent. In fact, they may have partly opposite impact on network organization. The processing efficiency constraint favors network shortcuts that link topologically distant parts of the network, which may take the form of long-distance connections, in which case they would act against the metabolic cost constraint of wiring length minimization. Conversely, wiring minimization favors the creation of links among spatially adjacent network nodes, which may also be topological neighbors (cf. Fig. S3 in [63]). Such networks with mainly local connections typically possess low path efficiency due to a large average number of processing steps. In conclusion, the two constraints need to be considered in combination. The coexistence of modular organization and hubs in networks could be, at least partially, understood by a balance of these two constraints. Indeed, there is growing evidence to support the idea that neural network connectivity is not optimized either to minimize connection costs or to maximize advantageous topological properties, but rather is an economical trade-off between the physical cost and the adaptive value of its topology [88], see [52] for a recent extensive review. It has been proposed that the cost-efficiency balance of the human functional network [89] may be related to the behavioral performance in cognitive tasks [79], but the anatomical mechanism underlying such desirable functional connectivity is not clear.

In the present work we explored the relationship between multiple constraints and complex network architecture by systematically testing the effect of the competition of multiple constraints. We considered the neuronal network of *C. elegans* [63], [65], [90], [91] and the cortico-cortical network of the non-human primate (Macaque) brain based on tract-tracing studies [63], [92], for which information is available for both the spatial positions of the nodes and the network connectivity. In a previous analysis of the Macaque cortical network [27], [63], the division of the motor areas was not very highly resolved, which feature might induce biased results when analyzing network modules and spatial clustering. Therefore, in the present work we first improved the data set by a more detailed division of the motor areas based on the CoCoMac database [92], extending the former 6 motor areas to 15 areas with an additional 128 connections (see *Materials and Method* and Fig. S1 for the adjacency matrix). Different from the CPO method, in our scheme we compared the real network connectivity to reconstructed networks derived from multiple constraints by fixing the spatial position of each network node and the total number of (directed) connections as in the real networks. The reconstructed networks were obtained under various balancing conditions of wiring cost and processing efficiency constraints. As in previous studies [53]–[55], [57]–[66], we used the total physical distance of the wiring 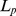 to represent the effect of the wiring cost constraint, and the total graph distance of the shortest paths 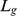 to represent the influence of the processing efficiency constraint, and defined an objective function 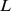 as a combination of both constraints using a weight parameter 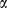, namely, 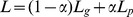, with  and 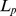 appropriately normalized. So 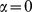, or 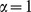 corresponds to a single constraint of path efficiency or wiring cost, respectively. Then we reconstructed the connections of the network with the help of a simulated annealing approach to minimize the objective function 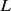 at different values of 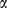, starting from 50 random configurations (see *Materials and Methods*). We studied the general properties of the competition between the two constraints in a 1D model with one-dimensional uniform spatial layout of nodes and directed connections. For the real neural networks, we investigated the modularity and hub properties of the reconstructed networks and studied the relationship to the spatial layout and compared them to those of the real networks. We found that for certain intervals of balancing these two constraints, the reconstructed networks showed a very similar modular structure and similar spatial positions of the hubs as the real networks. These results are also related to the nonuniform layout and clustering of the network nodes (neurons for *C. elegans* and areas for Macaque cortical brain) in space. Despite the observed agreements, there still exists significant discrepancies between model and real networks, suggesting that there are additional functional requirements to be considered in the future.

## Results

### Competition between cost and efficiency constraints leads to the coexistence of hubs and local connections

**Figure 1:**
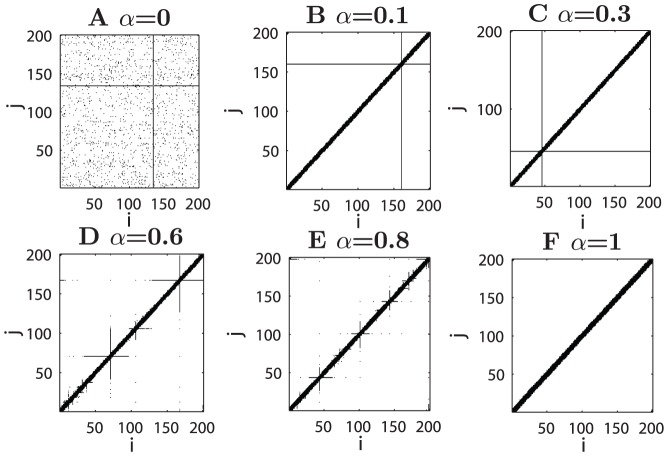
Constructed networks in a 1D model. There are 200 nodes uniformly placed on a one-dimensional circle linked by a total of 2000 directed connections. Shown are the adjacency matrices obtained at various 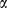 values, as indicated on the top of the plots. The nodes are indexed by their locations on the circle, common for all panels. (A) 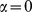, (B) 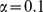, (C) 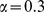, (D) 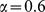, (E) 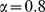 and (F) 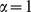.

**Figure 2:**
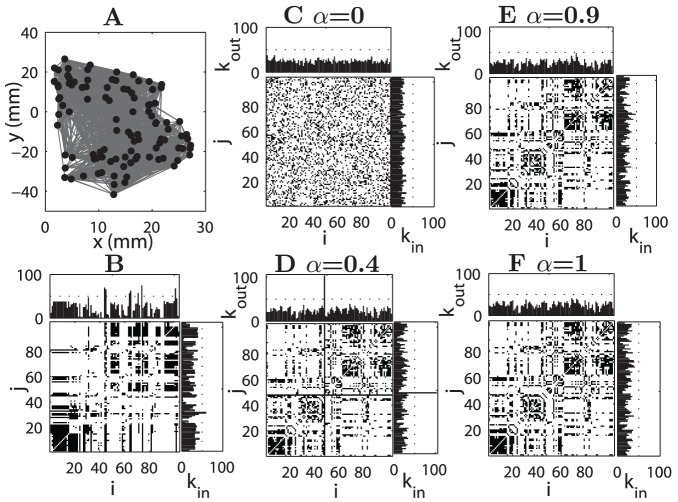
Comparison of reconstructed and original connectivity of Macaque cortical network. The left two plots (A and B) are for the original network. (A) Layout placement of 103 areas and connections between them. (B) Adjacency matrix, the output (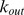) and input (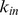) degrees of the areas. The right four plots (C–F) show adjacency matrices and the degrees of areas in the reconstructed networks at various values of 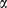. (C) 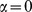, (D) 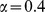, (E) 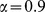 and (F) 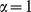. The index of the cortical areas is the same for (C–F) and the names of the areas are listed in Table S1 of Supporting Information (SI).

**Figure 3:**
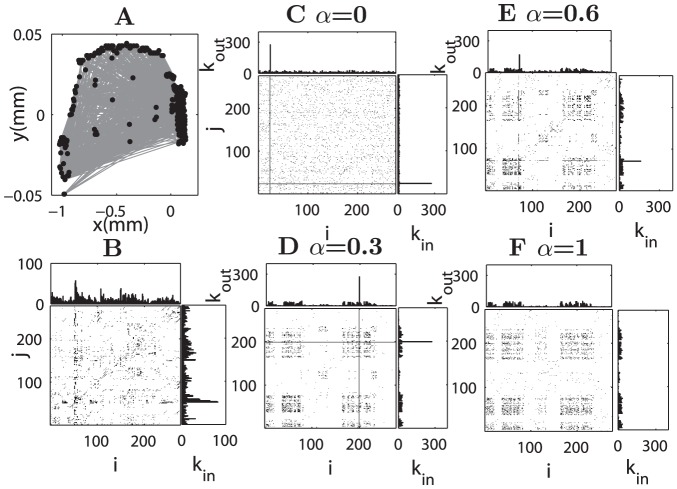
Comparison of reconstructed and original connectivity of *C. elegans* neuronal network. The left two plots (A and B) are for the original network. (A) Layout placement of 276 neurons and connections between them. (B) Adjacency matrix and the output (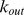) and input (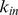) degrees of the areas. The right four plots (C–F) show adjacency matrices and the degrees of areas in the reconstructed networks at various values of 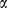. (C) 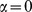, (D) 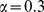, (E) 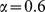 and (F) 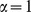. The index of the neurons is the same for (C–F) and the names of the neurons are listed in Table S2 of SI.

The qualitative properties of the competition between the constraints at different values of 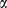 were found to be quite common for the 1D model (Fig. 1), the real Macaque cortical network (Fig. 2) and *C. elegans* neuronal network (Fig. 3), the latter two having highly non-uniform spatial layout of nodes. These properties can be most clearly seen in the 1D model. In addition to 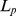 and 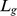, we used several parameters such as the number of hubs 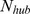, the average degree of the hubs 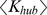 and the fraction of spatially local connections 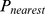 to characterize the reconstructed optimal networks at various 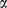 values (see *Materials and Methods*). The results are summarized below.

At 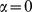, the network is only optimized by the processing efficiency constraint and achieves minimization of the topological distance. The optimal network configuration depends on the density of connections. If the initial network is sparse (roughly, connection probability 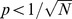), as in the 1D model (, Fig. 1A) or *C. elegans* neuronal network (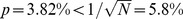, Fig. 3C)), the reconstructed network is characterized by a single hub connecting to all other nodes while the rest of connections appears to be random. The position of the hub node is arbitrary. A single hub is very effective for reducing the graph distance when the network is sparse enough, because the path length between any pair of nodes is either 1 (direct link) or 2 (connected through the hub). When the network becomes denser (), such as the Macaque cortical network (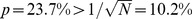), the pathlength between any pair of nodes in a random network cannot be larger than 2. It is then very unlikely to obtain a single hub when optimizing from an initially random configuration. Thus, the reconstructed network connections appear to be random (Fig. 2C). The dependence of the network configuration on the connection density is also more systematically shown in the 1D model (Fig. S2).

**Figure 4:**
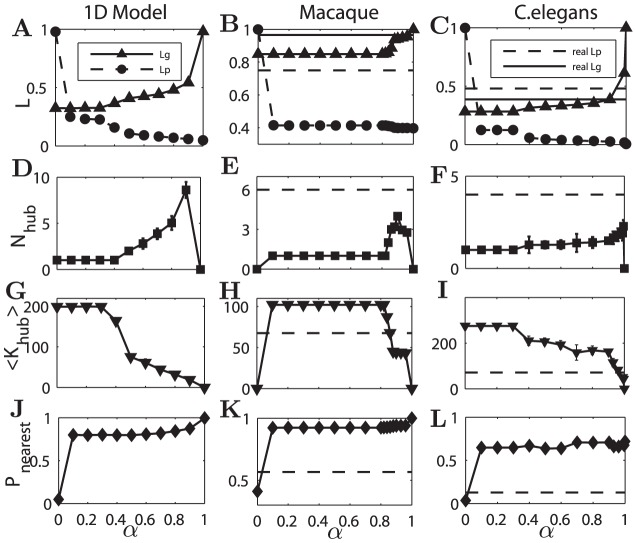
Properties of the reconstructed networks as functions of 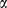
 **.** Results are shown for the 1D model (left panel), Macaque cortical network (middle panel) and the *C. elegans* neuronal network (right panel). (A, B, C) 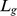 and 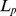 (normalized) of the reconstructed networks (with triangles or dots respectively), which are compared to those in the original networks in B and C. (D, E, F) 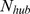, the number of hubs in the reconstructed networks vs. 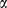. Here a node with z-score of total degrees (input and output) larger than 2 is considered as a hub. (G, H, I) The average degree of the hubs in the reconstructed networks. (J, K, L) The probability 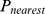 of connections to the spatial nearest-neighbors. The results are obtained by averaging over 50 realizations of the reconstructed networks for each 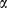. In most cases the error bars are smaller than the symbol size. The dashed lines are the corresponding results for the real network.

At 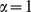, the processing efficiency constraint plays no role while the wiring cost constraint is fully dominant, and the network has the minimal total wiring length. Most of the connections are local and there is no hub (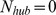) in any of the three systems (Figs. 1A, 2F, 3F). For the 1D model or the reconstructed networks of the Macaque, all the connections are local (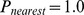, Fig. 4J,K); 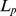 is reduced to 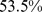 of that in the real Macaque network (Fig. 4B). As for the *C. elegans* network, many neurons in the head and tail are very densely distributed with very small distance among them, while the distance between neighboring neurons in the ventral cord is much larger. Therefore, significant numbers of non-nearest but short-distant connections within the head or the tail let the physical distance 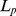 become very small, only 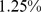 of the real *C. elegans* (Fig. 4C), but 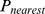 is clearly smaller than 1.0 (Fig. 4L)). And in the real networks, the spatial layout of the network nodes is non-uniform, forming spatial clusters; these local connections make the adjacency matrix become non-uniform, showing some clustered pattern similar to the original network connectivity (Figs. 2F, 3F). Spatial clustering and module organization in the reconstructed and real networks are studied below.

For 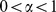, the processing efficiency and wiring cost constraints combine their impact, resulting in two distinct regimes, depending on the networks. When 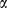 is positive, but not very large, such as 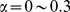 for the 1D model (Fig. 1B,C), 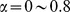 for Macaque (Fig. 2D) and 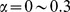 for *C. elegans* (Fig. 3D), there is a single hub connecting to all the other nodes in all the three systems (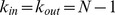). Most of the other connections are linked to the nearest neighbors, and 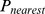 is close to 1.0 in the 1 D model (Fig. 4J) and the reconstructed networks of Macaque (Fig. 4K), but is clearly smaller in *C. elegans* for the reason stated above (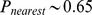, Fig. 4L). This single, global hub is a very effective configuration to provide high efficiency, when the other connections are short-distance due to the cost constraint. While the placement of the hub is arbitrary in the 1D model due to a symmetrical spatial layout (Fig. 1B,C), it is unique in the reconstructed networks of Macaque and *C. elegans*, located close to the global geographical center of the whole network, as will be discussed in detail below. In this regime, 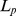, 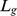, 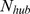 and 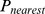 are all constant with respect to 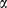, since the optimal network configuration does not change with 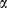, though the speed of convergence in the optimization process does. While both 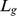 and 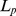 in the reconstructed networks are smaller than those in the real networks in this region, 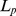 is much more smaller (reduced to 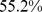 in Macaque and to 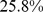 in *C. elegans*, see Fig. 4B,C).

The insensitivity of the optimal configuration to 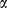 in this regime can be understood from the objective function 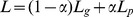. The variation is 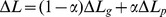, where 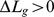 and 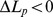 with increasing 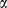. The network configuration would change with 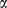 only if 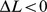, i.e., 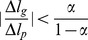. However, for the Macaque network, we found that 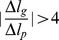 for any perturbation to the configuration obtained at 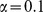 (a single global hub and all other local links). When 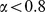, 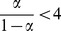, thus 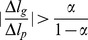, then 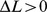, and the configuration change cannot be accepted by the optimization. Only when 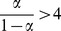 (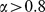), it becomes possible to have 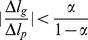 for certain perturbations, and a configuration change will happen. For this reason, in all the three networks, the optimal solution is the same in a range of 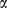 values, depending on the spatial layout of the network.

With further increase of 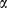, the influence of the wiring cost constraint becomes stronger. A single hub is no longer found, because very long-distant connections are prohibited and the systems move into a different regime (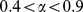 for the 1D model, 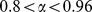 for Macaque and for *C. elegans* 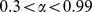). Several smaller hubs emerge, with connections extending to nodes in their spatial neighborhood, and the connection range of such regional hubs becomes smaller at larger 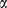, as can be most clearly seen in the 1D model (Figs. 1D,E), and is also true in the real networks (Figs. 2E and 3E). Consequently, 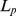 is further reduced slightly, but 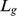 increases and is close to that of the real network (Fig. 4B,C). As shown below, the spatial positions of the hubs in the reconstructed networks are close to the real hubs in the two original networks.

When 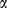 is very close to 1.0, where the efficiency constraint is weak while the wiring cost constraint is almost fully dominant, most of the connections are local and there are no pronounced hubs (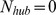). 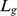 increases quickly and becomes larger than that in real networks.

We also found that for all 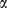 values, the input and output degrees of the nodes in the reconstructed networks are largely symmetrical (Fig. S3A and Fig. S3C). While the input and output degrees were found to be significantly correlated in the real networks (Fig. S3B and Fig. S3D), the discrepancy between the optimization model and real data is quite large, because this model does not include possible requirements that could generate the asymmetry, for instance, input-output information flowing as in real networks (e.g., from sensory neurons to motor neurons in *C. elegans*).

The above results show that the coexistence of local connections and hubs in the cortical networks could be a solution to the multiple constraints of wiring cost and processing efficiency. There is a regime (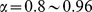 for Macaque and 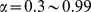 for *C. elegans*) in which the competition of the constraints can allow the formation of several hubs connecting to many of the nodes in the neighborhood. Here, 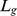 in the reconstructed networks is very close that of the real networks, but 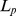 can be much smaller. In the following sections, we show that both the modular structure and the positions of hubs are quite similar to the real networks in this regime.

### Modules in network connectivity and spatial clustering of nodes

The above results (Figs. 2D–F and 3D–F) showed the emergence of a modular organization in the reconstructed networks similar to the real ones, which is derived from the non-uniform spatial distribution of the nodes and the local connections due to the constraints. In order to further explore these relations, we examined the relation between the spatial clustering of nodes and the modules in network connectivity both in the real and reconstructed networks, and compared the similarity between the modules in the real and reconstructed networks.

#### Macaque cortical network

**Figure 5:**
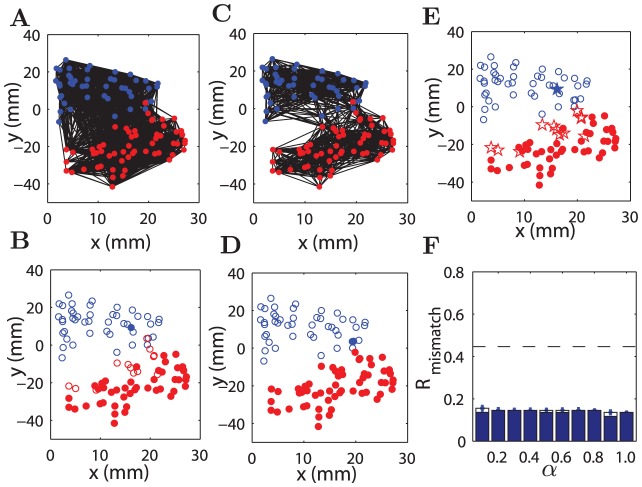
Modularity of original Macaque network and reconstructed networks. (A) Layout placement of 103 areas and the connections of the Macaque cortical network. (B) The two modules of the real network (open and filled circles) are compared to the two spatial clusters (blue and red). The corresponding modularity is 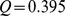. (C) As in (A), but for a reconstructed network at 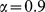. The blue and red colors of the nodes represent the two spatial clusters. (D) The same as (B), but the modules are from the reconstructed network in (C), with 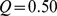. (E) Mismatch between the module partitions of the reconstructed and real networks. The mismatching areas are indicated by the pentagrams. (F) 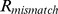 shows the fraction of mismatching areas between reconstructed and original networks in module partition, with respect to 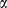. The result did not include 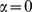 where the reconstructed networks did not show strong modularity. Blue bars are for the mismatching areas appearing in more than 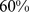 of all 50 realizations at each 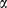, while white bars are for the mismatching areas appearing at least once in the 50 realizations at the given 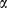. The dashed line represents the mismatching rate between the real networks and shuffled modules (see section “Matching between partitions” in *Materials and Methods*).

In the the real Macaque cortical network, the nodes (which represent the centers of mass of the cortical areas) are distributed non-uniformly in the three-dimensional space due to size variations of the areas. Visual inspection of a two-dimensional layout in Fig. 5A suggested that the nodes could be divided into two spatially separated clusters. This was confirmed by clustering analysis (see *Materials and Methods*), shown by different colors (blue and red) of the nodes in Fig. 5A. Such spatial clustering was found to be quite robust in data sets with different parcellations of the areas. The clustering of this more highly resolved data set with finer division of the motor areas (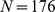) was compared with a parcellation of 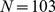 areas [93] (see *Materials and Methods*) and was found to be very similar. The clustering boundaries of the two data sets differed only by 5 areas (referring to the 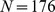 dataset) (see Fig. S4 and S5). Unfortunately, network connectivity is not available for this finest parcellation of 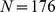 areas. We also examined the spatial density of the nodes within a radius around a given node, which we called the *neighborhood density* (see *Materials and Methods*), and found that it was also quite non-uniform due to heterogeneous area size. As shown in the following sections, many properties of the reconstructed and original networks of the Macaque cortex are related to these properties of the spatial layout of the network nodes.

The connectivity in the original Macaque cortical network shown in Fig. 5A also suggests that the connections are denser within spatial clusters than between clusters, indicating that the spatial clustering of the areas and densely connected network modules are related to each other. We analyzed modules in the original Macaque cortical network and the reconstructed networks (see *Materials and Methods*). The optimal modularity is 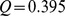 in the original Macaque network (Fig. 5A), corresponding to two modules shown by open and filled circles in Fig. 5B. The modularity did not show uncertainty with respect to the modular boundaries and is quite significant when compared to the corresponding random networks with the same number of nodes and connections (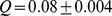). We can see that the modules and spatial clusters have strong overlap, and only 14.6% of the total nodes are mismatched. An example of a reconstructed network (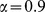) is shown in Fig. 5C, which has much fewer connections between the two spatial clusters than the real network in Fig. 5A, therefore has a larger optimal modularity 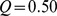 (also two modules, without uncertainty). The two modules from the reconstructed networks overlap almost completely with the two spatial clusters, respectively (Fig. 5D), with only one mismatched areas (0.97%). We examined the mismatched areas between the module partitions of the original and the reconstructed network at various 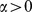 and found that they were consistent. The number of the mismatched areas remains small (around 15% of all the areas, much smaller than the mismatching rate for real networks and shuffled modules (see *Materials and Methods*) for all 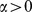, but is clearly smaller for 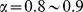 (blue bars in Fig. 5F). The modularity 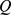 of the reconstructed networks was larger than that in the real network for 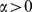 (see supplementary Fig. S6A). Note when 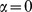, the reconstructed solution under the single efficiency constraint is a random-like network without clear modular structure (modularity Q = 0.096
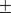
0.005).

#### *C. elegans* neuronal network

**Figure 6:**
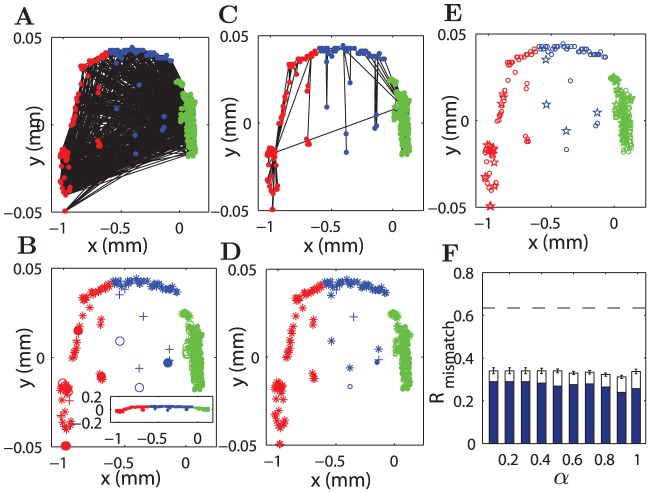
Modularity of original *C. elegans* neuronal network and reconstructed networks. (A) Layout placement of 276 neurons and the connections of the network. (B) The four modules of the real network (open, filled circles, plus and asterisk) are compared to the three spatial clusters (blue, green and red). The corresponding modularity is 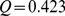. (C) As in (A), but for a reconstructed network at 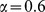. The red, blue, and green colors of the nodes represent the three spatial clusters. In (A) and (C), we used different scales for the 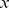 and 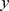 axis for clear presentation. The connections among dense nodes cannot be seen. The inset of (B) shows the positions and clusters of neurons in identical scales. (D) The same as (B), but the modules are from the reconstructed network in (C), with 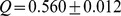. (E) Mismatch between the module partition of the reconstructed and real networks. The mismatched areas are indicated by the pentagrams. (F) 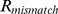 shows the ratio of mismatched areas between reconstructed and original networks in module partition, with respect to 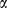. The result did not include 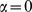 and 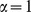 where the reconstructed networks did not show strong modularity. Blue bars are for mismatched areas appearing in more than 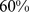 of all 50 realizations, while white bars are for mismatched areas appearing at least once in the 50 realizations for a given 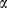. The dashed line represents the mismatching rate between the real network and the shuffled modules.

The spatial layout and connectivity of the real C. elegans neural network are shown in Fig. 6A. There are four network modules, with modularity 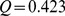 (Fig. 6B, different symbols). Again, there was no uncertainty in the module partition, and the modularity is significant compared to the corresponding random networks (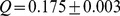). Using the spatial clustering method, the neurons were grouped into three clusters (shown by different colors). Interestingly, the relationship between connectivity modules and spatial clusters is similar to the Macaque cortical network. While not perfect, there exists some overlap between modules and spatial clusters (Fig. 6B), about 30.8% of the neurons are mismatched. For the reconstructed network of *C. elegans*, quite similar to the Macaque cortical network, there are fewer connections between different spatial clusters (Fig. 6C, 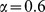) when compared to the real network, consistent with a larger modularity 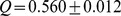 for four modules. The overlap between the modules and the spatial clusters is stronger than in the real network (Fig. 6D), and the ratio of mismatched neurons is reduced to 11.96%. The number of mismatched neurons between the modular partitions of the original and the reconstructed networks remains small (about 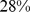 of all neurons) for 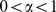, approaching the smallest value (nearly 23%) when 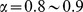, but increasing clearly for 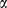 very close to 1.0 (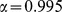) (blue bars in Fig. 6F). Again the mismatching rate is much smaller than that between the real network and shuffled modules. The modularity 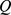 of the reconstructed networks is larger than that of the real network for 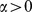 (see supplementary Fig. S6B). Note that for the singular constraint of path efficiency at 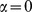 or of wiring cost at 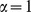, the reconstructed networks do not exhibit evident modularity (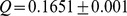 and 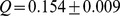, respectively).

We would like to emphasize that the results reported above have already taken the uncertainty of module partition and spatial clustering into consideration (see *Materials and Methods*). First, the uncertainty in the module partition is quite small. The Macaque cortex did not show degeneracy in module partition when (the toolbox of brain connectivity) BCT method was applied for many times, although degeneracy was indeed observed in a corresponding random network with the same number of nodes and connections (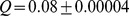, here the error-bar is from 50 applications of the BCT method). The real network in *C. elegans* did not have degeneracy either, but there was a small uncertainty in the reconstructed networks (with a typical fluctuation of 1–5 neurons from run to run). Second, we used 8 different distance measures to detect the hierarchical clustering. When comparing the clustering partition from these 8 methods to the modular division, the partition based on inner squared distance generated the minimal set that matched the module partition. The rest of the nodes formed the largest possible mismatched group that covered all the mismatched groups from different clustering methods (see *Materials and Methods*). In particular, there were 89 cortical areas in the Macaque which match between the modules and the clusters from all the 8 methods; the remaining set of 14 areas is thus the largest possible group covering all the unmatched groups across different methods of clustering and was reported in Fig. 5B (among these 14 unmatched areas, 5 areas appear to be the unmatched areas in all these 8 methods, 1 area in 7 methods, 6 areas in 5 methods, and the 2 areas in 3 methods). In the real network of *C. elegans*, this set of most stable nodes contains 191 neurons and the remaining 85 neurons are considered as the the largest group of mismatched neurons (Fig. 6B). In the same vein, the reported mismatching between the module partitions of real and reconstructed networks is the largest possible group when taking the uncertainty into consideration (Figs. 5E,F, and 6E,F).

These results show that the modular structure of the original networks is strongly related to the fact that the network nodes are spatially clustered, and many of the connections are contained within the clusters due to the effect of the wiring cost constraint. However, the best match of the modular partition with the real network involves the processing efficiency constraint (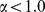) that enforces some long-range connections between the clusters.

### Location of hubs

The results in Section I. showed that the emergence of hubs could be the result of the combination of the wiring cost and path efficiency constraints, since hubs connecting to many nodes are very effective for improving path efficiency, while allowing most of the other connections to remain local to satisfy the cost constraint. If the hub is a global one, connecting to all the nodes, then it is reasonable to expect that the position of the hub is not arbitrary, but is near the geographical center (the node with minimal total distance to all the other nodes) of the whole network in order to maximally reduce the total wiring length of the connections from the hub. If the hub is a regional one, connecting to most of the nodes within the local region (e.g., within one of the spatial clusters), then the position should be close to the geographical center of this region. Thus, a node with many other nodes densely distributed in the neighborhood (having a high neighborhood density, see *Materials and Methods*), could be an candidate for a hub under the two constraints.

In the following section, we present findings regarding the location of the hubs in the reconstructed as well as the real networks. In our analysis, network nodes with z-score of total degrees (input and output) larger than 2 (see *Material and Methods*) are considered as hubs. The locations of the hubs in all 50 realizations of the reconstructed networks at each 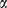 were identified. We would like to point out that identifying hubs in this way is heuristic and may introduce some ambiguity when comparing different networks (real or reconstructed at different 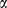). However, hubs that were defined in this way indeed provided a plausible way to describe the variation of the nodes with the largest degrees in the network as 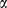 changes.

#### Macaque cortical network

**Figure 7:**
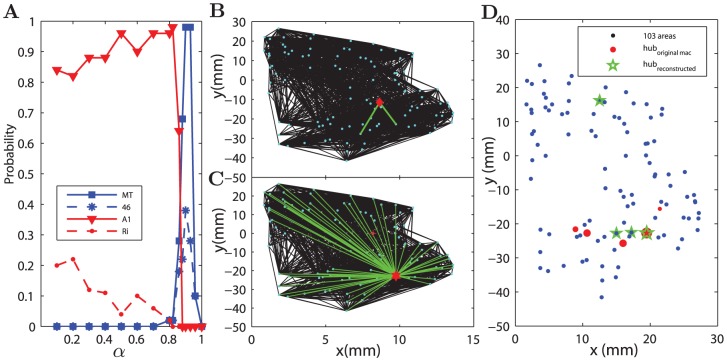
Hubs in the reconstructed and original Macaque networks. (A) Four areas A1, Ri and V5/MT and area 46 had the large probabilities to appear as one of the hubs in the reconstructed networks at different 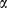. Note that V5/MT is also the biggest hub and area 46 is the biggest out-degree hub in the original network. (B) The position and connections (green lines) of the area A1 (global geographical center) in the real network. (C) The position and connections of the biggest hub V5/MT in the real network. (D) 6 hubs in the real network (red bullets, with the size of the symbol indicating the total degree) and 4 hubs (V5/MT, area 46, MSTm, MSTd) in the reconstructed network at 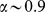 (green stars). The positions of the reconstructed or real hubs coincide or are close to each other.

For the Macaque cortical network, there was only one global hub connecting to all the other areas in all optimally reconstructed networks for 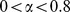. Either the primary auditory area A1 (the area index No. 41) or the area Ri (No. 73) was selected as the single hub, with larger than 80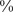 and smaller than 20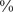 probabilities, respectively (Fig. 7A). When checking the global geographical centrality (see *Materials and Methods*), we found that A1 and Ri were ranked No. 1 and No. 2, respectively, which is consistent with our expectation. However, in the real network these two areas are non-hub nodes, with only 2 connections for A1 and 21 for Ri. The position and connections of A1 in the real network are shown in Fig. 7B.

When 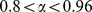, the number of hubs increases while their average degree decreases, and the hubs become regional. Further increasing of 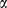 to be very close to 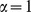 prevents the emergence of hubs, because the impact of the wiring cost constraint becomes too strong relative to the influence of the processing efficiency constraint. For 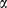 around 0.9, four areas, V5/MT, area 46, MSTm and MSTd appeared as hubs in many realizations of the reconstructed networks (Fig. 7D, green stars). It is important to note that area V5/MT and area 46 are respectively the biggest hubs in the original network in terms of total degree and output degree (these two areas are also identified as hubs in [20]). V5/MT has a total of 112 input and output links, with 87 of them from the red spatial cluster (connecting to 87.9% of nodes in this cluster) and 25 from the blue cluster. The location of area V5/MT and its all connections in the real network are shown in Fig. 7 C. Although V5/MT is only ranked No. 27 in terms of the global geographical centrality, it is ranked No. 3 of the regional geographical centrality of the red cluster. It is also ranked No. 1 in terms of high neighborhood density in a range of radius (specifically, 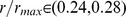, see *Material and Methods*). The biggest output hub area 46 has 59 efferent links in the real network. It is ranked No. 2 of the regional geographical centrality of the blue cluster and No. 3 by neighborhood density in a range of radius 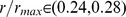.

In the reconstructed networks with 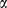 around 0.9, V5/MT and area 46 were respectively selected as hubs in 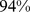 and about 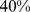 of the realizations (Fig. 7A). The other two areas MSTm and MSTd were selected as hubs in the reconstructed networks, because they are respectively ranked No. 1 and No. 2 in terms of the geographical centrality of the red cluster and No. 2 and No. 4 by neighborhood density. But these two areas are not hubs in the real network.

Apart from the biggest hub area V5/MT, there are five more hubs (z-score above 2.0 either in total, input or output degree) in the real Macaque cortical network, areas 7a, VIPl, 7b, LIPv and area 46. All the hub areas are shown by the red bullets in Fig. 7D, with the size of the symbol indicating the total degree. Except for area 46 located close to the geographical center of the blue cluster, the other five hubs are all located within the red cluster and are among the top 18.5% in terms of the regional geographical centrality of this cluster. The ranking of a few nodes with the largest total degrees in terms of neighborhood density and geographical centrality is also shown in Fig. S7. The positions of the real hubs thus coincide or are close to those hubs in the reconstructed networks which are located in the regional geographical centers.

#### *C. elegans* neuronal network

**Figure 8:**
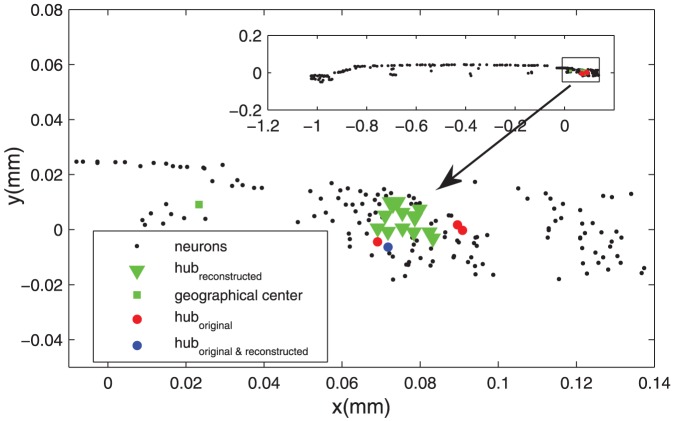
Hubs in the reconstructed and original *C. elegans* networks. The inset shows the two-dimensional layout of all 276 neurons of *C. elegans*. The square region in the inset is zoomed out, showing the four hubs in the real network (red and blue bullets), the global geographical center (green square) and the hubs in the reconstructed networks (green triangles) and the overlap with the real hub AVBR (blue bullets).

For the real *C. elegans* neuronal network, four neurons were identified as hubs (z-score above 2.0), which are AVAL(left)/AVAR(right) and AVBL/R (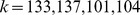), all located in the head (red bullets in Fig. 8). These 4 hubs are among the top 27.2% in terms of the regional geographical centrality of the head. Note that the neuron FLPL having the highest global geographical centrality of the whole system (red square Fig. 8) is also in the head, as most of the neurons (166 out of 276) are in the head.

In the reconstructed network, the global geographical center FLPL was indeed taken as the single hub connecting to all the other neurons for 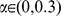. With stronger impact of the wiring cost constraint for 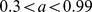, the number of hubs increases while their average degree decreases. In total, 14 neurons were chosen as hubs (with a probability above 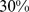 to appear in the reconstructed networks at certain 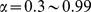), with degree ranging from 140 to 200, connecting 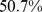 to 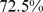 of the neurons. 10 of them were from 5 groups with left and right symmetry, ADFL/R, ASEL/R, ASHL/R, AWCL/R and RMHL/R, and the other 4 neurons were AIBR, RIBR, SIBDR and SIBVL. Interestingly, these hubs in the reconstructed networks (green triangles, Fig. 8) were close in location to the 4 real hubs (red bullets, Fig. 8). In fact, these 14 neurons are among the top 8.33% in terms of the regional geographical centrality in the head. Notably, we also found an overlap of reconstructed with real hubs. When 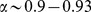, the real hub AVBR has a relatively small probability (24%) to be chosen as the hub in the reconstructed network (marked as the blue bullet in Fig. 8).

One may wonder whether it is meaningful to say that the hubs were spatially close when most neurons are located in the tiny head of the worm. Therefore, a more quantitative comparison is desirable to support the visual inspection. We calculated the sum distance for each of the 166 neurons in the head to the 4 real hubs and sorted the neurons in the order of increasing distance (Fig.S8). It is clear that most of the neurons taken as hubs in the reconstructed networks are among the closest neurons to the real hubs.

While generally there is ambiguity in defining hubs in the networks, our observation is clear: with stronger influence from the wiring cost constraint, the reconstructed networks shift from a configuration with a single global hub to configurations having several nodes with relatively large degrees, and their spatial locations move from the global geographical center to the regional geographical centers accordingly. In real networks, the nodes with the largest degrees are close to the regional geographical centers, showing that both the processing efficiency and cost constraints are at work. This finding is strong evidence to support the hypothesis of a trade-off, though it appears there are more nodes with larger degrees in the real networks than the reconstructed networks.

### Degrees of nodes and correlation with neighborhood density of nodes

The results in the above sections suggested that the mesoscopic properties of networks, the simultaneous formation of modules and hubs, can be partially explained by the combination of the wiring cost and processing efficiency constraints. Now we examine the degree of nodes in the original and reconstructed networks.

**Figure 9:**
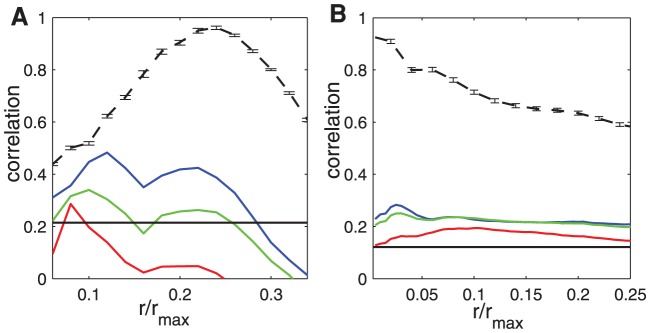
Degrees of nodes in the reconstructed and real networks. (A) and (B) Correlation between degrees and neighborhood density vs. the normalized radius 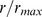 for reconstructed (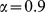) and real networks. (A) for Macaque and (B) for *C. elegans*. The results differ for the output (blue line), input (red line) and total degree (green line) in the real network, but are almost the same in the reconstructed network (black dashed line). The horizontal black line represents 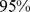 of significance level. The error-bar is from 50 realizations of the reconstructed networks.

When constructing networks under the wiring cost constraint, the nodes tend to connect to their nearest spatial neighbors, which is confirmed by a high value of 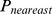. Therefore, the number of connections of a node (degree) is expected to be related to the neighborhood density of the node in a certain spatial range. We calculated the density of nodes for various radii 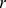 and evaluated its correlation with the degree of nodes. The correlation between the degrees and density in reconstructed network with strong enough wiring cost constraint (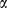 close to 1) is quite large in a range of 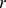 for both the Macaque and *C. elegans*, as shown by the black dashed curve in Fig. 9A,B.

Interestingly, the correlation between degrees and density is also significantly present in the real network (colored curves in Fig. 9A,B). For example, the correlation can reach 0.49 at 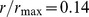 for Macaque and 0.29 at 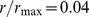 for *C. elegans*, much larger than the 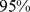 of the significance level in the corresponding surrogate data. The results are consistent with the observation above that hubs in the real network are ranked high in term of the neighborhood density.

Although the correlation between degree and neighborhood density is significant in both real and reconstructed networks, the discrepancy is also quite large. In particular, the degree distribution in the real network was not well reproduced in the reconstructed network (see Fig. S9 for a comparison). In both neural systems, the real networks have higher probabilities to have large-degree nodes, but the reconstructed networks have higher probabilities to generate intermediate-degree nodes, because the connections in the reconstructed networks are much more strongly determined by the neighborhood density. It has been shown that both the in- and out- degree of the *C. elegans* neuronal network obey a power-law distribution [47]. Our test of the significance of the power-law fitting to the distribution (see *Materials and Methods*) confirmed this statement (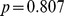, 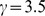, 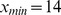 for in-degree and 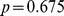, 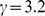, 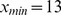 for the out-degree, Fig. S9). The real Macaque cortical network and the reconstructed networks of both systems do not show scale-free features. For all these networks, the largest 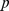 value is 0.02
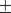
0.02 for the out-degree distribution of *C. elegans* obtained at 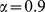.

These results indicate that degrees of the individual nodes reflect the impact of the wiring cost constraint, however there are still some other unknown factors in addition to the wiring cost and processing efficiency constraints that may strongly influence the node degrees in the original network.

### Link recovery

In the above sections we explored the similarity between reconstructed and real networks in global network measures (e.g., physical and graphical distance), and in mesoscopic properties (e.g., modules, hubs, degree and degree distribution). Next we analyzed how well the reconstruct networks recovered the connections in the real network. We calculated the recovery rate 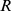 by comparing the overlap of the adjacency matrices of the original and reconstructed networks entry by entry, taking both asymmetry of 0's and 1's entries and the directionality of connections into consideration (see *Material and Methods*). 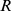 is maximal at 1.0 when two networks are identical in all connected and un-connected pairs.

**Figure 10:**
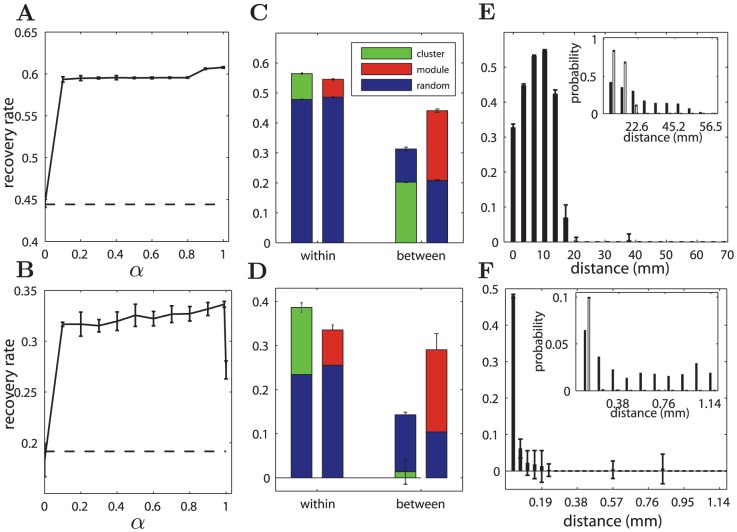
The rate of connection recovery in the reconstructed networks. The upper panel is for the Macaque cortical network and lower panel for the *C. elegans* neuronal network. (A, B) The recovery rate 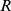 as function of 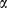. (C, D) Recovery rates among the nodes within the two spatial clusters and between the clusters (green bars for the reconstructed networks), as well as within and between modules (red bars for the reconstructed networks). The blue bars are the recovery rate of random networks with the same input and output degrees as the real networks. (E, F) The recovery rate as function of the spatial distance of the connections in real networks. The insets show the probability of connections as function of the spatial distance between two nodes, in real networks (dark bars) and reconstructed networks (white bars). The results in (C,D,E,F) are for 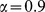 for both Macaque and *C. elegans*. The error-bars are from 50 realizations of the reconstructed networks.

The overall recovery rate 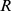 of the whole network as a function of 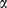 is shown in Fig. 10A,B for the two neural systems. For the Macaque cortical network, when 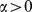, the recovery rate is nearly 60% (Fig. 10 A), which is clearly larger than the recovery rate of random benchmark networks obtained by rewiring the original network while retaining the input and output degrees. For the *C. elegans* neuronal network, when 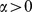, the recovery rate is more than 30%, which is significantly larger than 19.14% of random benchmark networks (Fig. 10 B). The recovery rate is much smaller than that of the Macaque networks, partially due to much sparser connectivity in *C. elegans*.

Importantly, the recovery rate is not uniform in the networks. It is higher within the spatial clusters, but lower between the clusters, as shown as green bars in Figs. 10C, D for Macaque and *C. elegans* respectively (both for 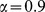). Notably, for *C. elegans*, the recovery rate of the connections within clusters could approach 40% (Fig. 10 D), almost doubling that of the random networks (blue bars). For the connections between the clusters, the recovery rate is even smaller than that in the random benchmark networks in both systems, especially significant for *C. elegans*, because the connections between clusters become too sparse in the reconstructed networks (see Figs. 5C, 6C). The real neural network has more long-range connections between spatial clusters, likely due to additional functional requirements.

Although the recovery rate within modules (red bars) is also larger than that of the random networks, particularly within spatial clusters, the recovery rate between modules is much higher than for random benchmarks for both systems, in contrast to the case between spatial clusters. The different results between clusters and between modules originate from the mismatching of modules and clusters.

In the combination of wiring cost and efficiency constraints, most of the connections are local. Therefore, the recovery rate is expected to be higher for the local connections. Our more detailed analysis of the recovery rate for the connections of different physical distance confirmed this expectation (Fig. 10E,F). In both the reconstructed and real networks, the probability of two nodes to be connected decreases with distance (insets, Fig. 10E,F). However, in real networks, the connection probability for very short distance is much smaller than in the reconstructed network, but it decays more slowly with the distance; therefore there is much higher probability of long-range connections, especially in *C. elegans*, likely due to other functional requirements. Nevertheless, the recovery rate is much higher for short-distant links, around 50% in both systems.

There are some differences between the *C. elegans* neuronal network and Macaque cortical network. (1) The *C. elegans* neuronal network is quite sparse (connection probability 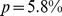), while the Macaque cortical network is rather dense (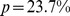). Therefore the recovery rate of the random benchmark networks is smaller in *C. elegans* (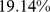) than Macaque (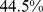). Furthermore, when 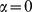 (efficiency constraint only), the reconstructed networks of *C. elegans* possess a global hub, but for the Macaque network they are quite similar to random networks (Fig. 2C and Fig. 3 C). Thus for *C. elegans*, 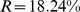 at 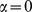 is even smaller than that of the random networks (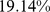). (2) About 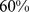 of the neurons in *C. elegans* gather in the tiny head, and the others scatter in the ventral cord and tail. This highly heterogeneous spatial distribution of neurons requires more long-distant connections in the real network. Indeed, *C. elegans* has a smaller fraction of local connections (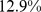) when compared to Macaque (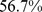) (Fig. 4K, L, dashed lines). These properties are perhaps the main reasons that the recovery rate in *C. elegans* is low, because too many short-distant links are put into the small head in reconstructed networks when compared to the real networks (Fig. 6 and Fig. 10E,F).

## Discussion

### Summary

The formation of complex neural networks is subject to many structural and functional constraints. In particular, it has been speculated that the network organization is the result of an economical trade-off between the physical cost and the functional value of the topology [52]. Among various graph theoretical measures, the average shortest pathlength representing the processing efficiency can be taken as an essential representative of functional constraints. In this study, we showed in a systematic and quantitative manner that the competition between these two straightforward constraints, the overall wiring cost and signal propagation efficiency, plays an important role in the network organization of Macaque cortical connectivity and *C. elegans* neuronal connections. By reconstructing network connections using optimization under multiple constraints, while fixing the component layout as in the real network and comparing the properties the reconstructed to the real networks at different topological scales, we revealed that the connectivity in both neural networks is closely related to the spatial arrangement of the nodes. The main findings are as follows. *i*) The combination of the wiring cost and processing efficiency constraints can lead to the simultaneous formation of local connections and hubs. *ii*) When the spatial layout of the nodes is not uniform, but clustered as seen in the real networks, this combination will lead to the formation of modules and hubs. The modules have strong overlap with the spatial clusters and the hubs are located at global or regional geographical centers. *iii*) In certain regimes of competition between the two constraints, the reconstructed networks have a modular organization quite similar to that in real networks, and the positions of the hubs coincide or are close to the actual hubs. *iv*) The analysis also revealed that the degrees of nodes in the real networks are correlated with their neighborhood density, and the reconstructed networks can recover a significant portion of the individual links of the real networks, especially for short-distant connections. These observations support the idea of a trade-off between cost and functional values. The two constraints, however, cannot fully explain all the properties in the real networks. There are discrepancies in several important aspects: (1) There are significantly more long-range connections in both real systems when compared to the model. (2) The correlation between the individual degree and the neighborhood density in the real networks is much lower than in the optimization model. (3) The degree distribution in the real network is different from that in the reconstructed network. (4) The model cannot generate asymmetry in the input and output degrees as in the real networks. All these differences suggest that there are additional important factors influencing the formation of real neural networks. In the following sections we discuss these points in more detail.

### Real neural networks are not optimized solely to minimize wiring length or to maximize processing efficiency

It is clear that real networks are not solely optimized by topological efficiency, due to a large number of local connections. Neither are they optimized only for minimal wiring cost, even though this cost constraint is playing a significant role. Previous studies investigated whether the component placement layouts in the real neural networks satisfy the concept of wiring length minimization, by comparing the wiring cost of real networks to perturbed component placement while keeping the network connectivity fixed. The accumulated evidence showed that the wiring cost constraint indeed plays an important role [55], [58], [59], [61], [63], [65], [69]. However, for the Macaque cortical network and *C. elegans* neuronal network, previous work using CPO while fixing the network connectivity showed that the wiring length is not minimized [63]. Here we reconstructed the network connectivity while keeping the component placement layout as in the real network. Under only the wiring cost constraint at 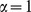, the reconstructed network differed significantly from the real networks: all the connections were short-distant and there were no hubs and no long-range connections as in the real networks.

The following comparison between real networks and reconstructed networks at 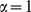 showed more clearly that the wiring cost constraint is at work, but the real neural networks are not solely optimized by minimal wiring length. (1) The wiring cost 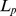 in the reconstructed networks is clearly lower than that of the real networks (53.5% for Macaque but only 1.25% for *C. elegans*), although the link recovery rate is still significant (60.8% for Macaque and 27% for *C. elegans*), since the wiring cost constraint does impose many short-range connections in the real networks. In the CPO solutions [63], by fixing the network connections as in the real networks, the wiring lengths were 68% and 52% of the real values of the networks in the Macaque and *C. elegans*, respectively. This finding shows that allowing hubs as in the real network is costly in wiring, especially for *C. elegans*. (2) The processing efficiency is much lower than in the real networks. As seen in Fig. 4B,C, 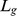 (the reverse of the processing efficiency) in the real networks is much smaller than the reconstructed networks at 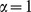. When 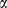 is slightly smaller than 1.0, the regional hubs in the reconstructed networks reduce the path length significantly and 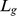 is close to the values in real networks; interestingly these values are not much larger than the optimal 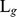 at 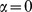. This finding is consistent with the previous observation that biological neural networks appear to be optimized for processing efficiency [63]. (3) The recovery rate is quite high within the clusters. In particular, the recovery rate of the connections within the Macaque visual system (covering 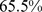 areas of the red cluster of Fig. 5) is 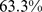, and it is 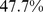 for the rest of the connections of the network, which is consistent with previous observations that the wiring among the Macaque visual cortex is relatively more wiring cost optimal than other cortical subsets [64]. (4) In *C. elegans*, solely minimizing the wiring cost at 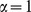 cannot produce strong modular structure in reconstructed networks (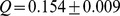, Fig. S6B) (as explained in the next section). However, similar modular organization as in the real network can be obtained under the balancing of two constraints (Fig. 3).

### Overlap between network modules and spatial clusters reflects the impact of the wiring cost constraint

We showed that the wiring cost constraint can predominantly determine the modular organization in real neural networks, and the partition of the modules is not very sensitive to the effect of the processing efficiency constraint at different 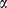. More precisely, the spatial clustering governs the connectivity modules in the reconstructed networks where most of the connections are extended to the spatial neighbors under the wiring cost constraint. Modules in the reconstructed network overlap largely with the spatial clusters. In the Macaque cortical network, the mismatched areas (mainly of the somatosensory system) are near the boundary of two spatial clusters (see Fig. 5E). This arrangement reduces the wiring cost when these areas make more connections to the other cluster to form a module, likely due to some further functional requirements.

Notably, the modular organization in the *C. elegans* neuronal network is most likely determined by the combination of the wiring cost and path efficiency constraints. With only the wiring cost constraint at 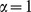, the reconstructed network of *C. elegans* did not show strong modularity as seen in the real network (Fig. S6B, 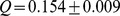 at 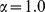). The main reason is as follows. About 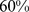 of the neurons gather in the tiny head, while other neurons scatter in the ventral cord and the tail with much longer physical distance between them. At 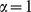, almost all the links in the reconstructed networks were put in the head to form a highly connected core. The rest of the nodes in the body and tail form an approximately one-dimensional array with a minimal number of necessary links in order to avoid disconnection from the main core, but do not form dense modules. However, when the path efficiency constraint becomes effective for 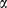 slightly smaller than 1.0, some long-range connections are forced between the hubs in the head and the other parts of the network. These long-range connections from the hubs now can also take the role of avoiding disconnection of the nodes into subsets. Now the remainder of the connections is allowed to be more short-ranged in the tail and in the ventral cord, which form modules that coincide with the spatial clusters. With suitable combinatorial influence of the two constraints at 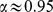, the fraction of mismatched neurons is reduced to about 23% (Fig. 6F).

In both systems, the best overlap between the modules in the reconstructed networks and real networks appears for a region 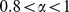, suggesting that the combination of the two constraints plays an important role in the formation of real neural networks which possess both modules and hubs.

### The processing efficiency constraint creates hubs by competition with the wiring cost constraint

The emergence of hubs can be attributed to the combination of the wiring cost and path efficiency constraints, because a single constraint on its own, either efficiency (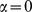) or wiring cost (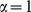), does not support hubs in networks as dense as the Macaque cortical network. For sparse networks, such as the one of *C. elegans*, the efficiency constraint on its own can generate a global hub, but the hub position is arbitrary. The wiring cost constraint is effective even for small 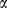 values. In both systems, there is a broad range of the competing parameter (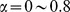 for Macaque and 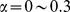 for *C. elegans*) where the reconstructed networks are composed of local connections and just one dominant hub. For larger 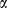, the wiring cost constraint becomes stronger and several regional hubs emerge, similar to the organization in real networks. However, the wiring cost constraint on its own (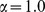) cannot enforce hubs in the reconstructed networks.

The nodes chosen as the hubs in the reconstructed networks occupy the highest ranks of the regional geographical centrality and neighborhood density in order to be wiring economic. Interestingly, real hubs are found close to these nodes with high centrality. Quite strikingly, in the Macaque cortical network, the location of the biggest hub at area V5/MT and the output hub of area 46 in the real network of the Macaque cortical network can be reproduced in the reconstructed networks by the combination of the wiring cost and path efficiency constraint at 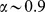. The strong impact from the wiring cost constraint in real networks is further reflected by the economic wiring of these hubs nodes: (1) the areas V5/MT and area 46 stay near the geographical centers of the red and blue clusters, ranked No. 3 and No. 2 of the regional geographical centrality of the red and blue clusters, respectively; these areas have most of their connections within their respective cluster. (2) V5/MT and area 46 are ranked No. 1 and No. 3 in terms of high density of nodes in the neighborhood within a certain range of radius. In *C. elegans*, the locations of hubs in the reconstructed network were also found to be very close to or overlapping with the position of real hubs, and there is also a regime (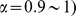 which best matches modules and hubs to those of the real network. In this regime, 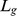 is close to the real networks, but 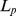 is much smaller, especially in the *C. elegans* neural network.

It appears in both systems that there are more nodes with larger degrees in the real networks than the reconstructed networks. However, importantly, the nodes with the largest degrees are close to the regional geographical centers similar to the model. Although one cannot expect that only two constraints can recover all the features of the real networks, the observation of the overlapping and close spatial locations of the reconstructed and real hubs (i.e., economic wiring of the hubs) provided clear evidence that probably both cost and efficiency constraints are at work in the real neural systems, supporting the idea of a trade-off between cost and functional values of the networks.

### Robustness against node failure might be an additional constraint

In a broad range of the balance parameter (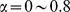 for Macaque and 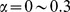 for *C. elegans*), the reconstructed networks have a single dominant hub linking to most of the nodes. Such a configuration is due to a strong impact of the path efficiency constraint, however, it is not functionally robust. Failure of the single dominant hub node greatly degrades the efficiency in information processing, because without the central hub, the network has mainly local connections. We performed a systematic analysis of the impact of removing the node with the largest degree in the reconstructed networks and measured the increase of 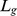 [94]. The results are shown in Fig. S10. It is seen that in the regime with a single, global hub, 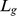 in the reconstructed networks increases significantly when the hub is removed. The degrading effect is much more serious in *C. elegans*, because the reconstructed networks without the hub contain an almost one dimensional array for the neurons in the body that separates head and tail. In the next regime with several regional hubs, (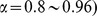 for Macaque and 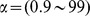 for *C. elegans*), the reconstructed networks are much more robust against the removal of the node with the largest degree (Fig. S10), very close to that in the real network. Thus, while hubs enhance the processing efficiency significantly, they are also the points of vulnerability to pathological damage [52]. The high energy consumption of the brain puts it under high vulnerability for energy undersupply, and the metabolically most expensive node is particularly vulnerable in pathological circumstances. There is evidence that metabolic costs of a node are proportional to its degree [95]; thus some brain disorders may be closely related to hubs' abnormalities. For example, this effect has been speculated to be a network mechanism for Alzheimer's disease [48], [52], [96]. In the *C. elegans* neuronal network, laser ablation of hub neurons (AVA and AVB interneurons) generated uncoordinated phenotypes [97], [98]. In particular, laser ablation experiments have demonstrated that AVA neurons are required for normal spontaneous and evoked backward locomotion [99]–[101], while AVB neurons are responsible for forward locomotion [100], [102]. Therefore, avoiding the configuration of a single dominant hub could indeed be functionally significant. In conclusion, the robustness requirement might be an additional important factor that generates the evolutionary pressure for the real networks to have more hubs, so that both in Macaque cortical network and *C. elegans* neuronal networks, the competition between the two constraints is settled down to a regime with 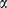 smaller, but very close to 1.0.

### Differences between reconstructed and real networks imply additional constraints

We have shown that there is a regime (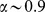) of the competition between the two constraints of wiring cost and processing efficiency where the reconstructed networks can reproduce some major properties of the real networks, including the simultaneous formation of hubs and modules, locating hubs or other large-degree notes close to the regional centers of the spatial clusters and similar resilience to node failure. However, there are still significant differences. The reconstructed networks have much smaller wiring length 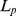 (about 54% of the real wiring length for Macaque and nearly 5% for *C. elegans*). This difference stems from the fact that the real networks have more long-range connections: there are more large-degree nodes or hubs (Figs. 4E,F, Fig. S3), the fraction of spatially local connections 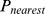 is much smaller and the probability of long-range connections is much larger (insets in Figs. 10E,F). Moreover, the number of connections is not strongly determined by the neighborhood density (Fig. 9).

Another significant difference is the asymmetry in the in- and out-degrees in real networks. Although directed links are used in our model influencing the calculation of processing efficiency, the objective function did not contain possible constraints that can reflect the functional role of the asymmetry in the input and output links; the reconstructed networks are thus highly symmetric. In fact, the asymmetry in the biological neural networks may be closely related to functional requirements of specific signal processing flow. For instance, in *C. elegans*, there are more connections from the sensory neurons to motor neurons than vice versa. Also from the connection matrix of the Macaque cortical network, there are more connections from some visual areas to the motor system, and fewer along the opposite direction. Therefore, in future work, one may include additional constraints to enforce information flow between different types of nodes, such as input nodes (the sensory neurons or primary sensory areas) and output nodes (the motor neurons or motor areas).

In our view, the differences imply that most likely real neural networks are influenced by additional functional requirements and constraints. The trade-off between the wiring cost and the processing efficiency with these new constraints could give a better account of the asymmetry and other properties of the real neural networks.

### Conclusion and outlook

We studied the formation of complex network connectivity derived from multiple constraints, in particular the competition between wiring cost and path efficiency requirements. By reconstructing networks while preserving the spatial layout of the components, we obtained an understanding of the relationship between the spatial layout and network connectivity derived from the multiple constraints. This understanding guided us to investigate the relationship between the spatial layout and network connectivity of the real Macaque cortical network and *C. elegans* neuronal network. The results are consistent with previous observations that wiring cost and efficiency constraints are playing an important role in shaping the network organization and provide evidence to support the idea of a trade-off between them. While significant, the wiring cost and path efficiency constraints cannot completely explain all the features in the connectivity patterns of the real Macaque cortical network and *C. elegans* neuronal network. Other factors, such as robustness of networks against node failure, more long-range connections and asymmetric input and output information flow appear to be important. Moreover, with our combinatory optimization model, we have mainly discussed the question of which constraints are at work to influence the neural network properties, but have not yet addressed possible mechanisms underlying the biological implementation of these constraints. Exploring additional functional constraints and incorporating them into growth or generative models [42], [103] may be worthwhile directions in the future research.

The present study on the impact of multiple constraints on the architecture of neural systems might also provide an anatomical foundation for the cost-efficiency balance in human functional networks [79]. A more detailed understanding of the relationship between the cost-efficiency balance in anatomical and functional neural networks will require the analysis and modeling of the dynamical interdependence in the reconstructed networks under different competition conditions relative to that in real networks.

## Materials and Methods

### Materials

#### Primate cortico-cortical network

We analyzed the connectivity of Macaque cortical network and its relationship with the three-dimensional spatial layout of the components and compared it to the reconstructed networks in order to understand the impact of multiple constraints. The Macaque network is largely based on a dataset with 94 cortical areas and 2,390 directed projections [63]. This data set is more suitable for our purpose compared to other connectivity data sets (e.g., cortical network of cat), since both the spatial positions of the components and the network connectivity are available. The connectivity data and three-dimensional spatial position (the average surface coordinate) of each cortical area were provided by M. Kaiser (http://www.biological-networks.org/) and amended with the help of the CoCoMac database (http://cocomac.org).

This dataset is extensive, but is still partly incomplete. Especially the divisions of motor regions are quite coarse with incomplete connections among several areas (e.g., the motor areas 4 and 6 cover a large territory, 6.5% of neocortex). Generally, it is a challenge to define anatomical parcellations that provide a high spatial resolution as well as a high density of the associated connectivity data. In this study we first improved the data set with a more detailed parcellation of the motor areas based on CoCoMac [92], which consists of three primary databases: literature, mapping, and connectivity. For the motor areas, there were 39 unique brain maps, 84 unique literature sets, and 454 unique records interrelating brain maps to each other. However, there are many types of inconsistency, including the lack of connectional information of some brain maps in the database, the supra-structure relations not being the symmetric transpose of sub-structure relations, typographical errors, brain regions with different acronyms, but with the same full name, etc. We checked the inconsistent and conflicting data. For instance, those maps lacking of connectional information in the connectivity database or with different acronyms but representing the same regions would not be included in the updated Macaque network. Those connections from the source sites A to the target sites B, if not consistent with the connections of region B receiving from region A, were removed. And connections between a motor area and different sub-structure areas of an existing area in the former database were merged. This expanded collation of data extended the former 6 motor areas to 15 areas (4, 4C, 6, 6D,6DC, 6DR, 6DS, 6m, 6Va, 6Val, 6Vam, 6Vb, SMA, SMAr, SMA-proper) with an additional 128 projections. Spatial positions of these areas were taken as the average surface coordinate, as in a previous approach [63]. This improved dataset of the Macaque cortical network has 103 areas and 2518 connections in total. The labels of the areas are listed in Table S1 of SI, and the adjacency matrix is shown in Fig. S1, where the new areas and links are highlighted by blue color. Although we applied a detailed dataset for the Macaque cortical network, the connectivity data may still be incomplete. We noticed that the motor region owns relatively low connection density (21.3%), comparing to the connection density of the whole Macaque cortex (23.7%). However, strong fluctuations of node degree and regional connection density have also been observed in most known connectivity data sets [35], [104], [105].

To examine the reliability of the spatial clustering (see “Spatial clustering” in *Materials and Methods*) of the components in this new dataset with 103 areas, we analyzed the clustering in another dataset -“PHT data” [93] with 176 areas from a finer parcellation of the subcortex and parts of the cortex in high magnification. The spatial positions of these areas were taken as the average surface coordinate estimated from surface parceling using the CARET software (http://sumsdb.wustl.edu/sums/index.jsp). However, connectivity information for this finer parcellation of areas is not available, and we only used the spatial positions of these areas for the spatial clustering analysis.

Spatial distance between the areas was calculated as Euclidean distance between the spatial positions of the areas, and the wiring length 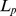 is the sum of the distances between connected areas.

#### *C. elegans* neuronal network

We also analyzed the connectivity of the *C. elegans* neuronal network and its relationship with the two-dimensional spatial layout and compared to the reconstructed networks. The dataset of the spatial positions of 276 neurons was provided by M. Kaiser (http://www.biological-network.org). The connectivity in this dataset is not complete. Thus, we compared the databases by Varshney et al. [47] and by Oshio et al. [91] on online neuronal wiring of *C. elegans* (http://www.wormatlas.org/neuronalwiring.html), and added those links that were common in these two new databases but did not exist in the dataset of Kaiser. Thus the original database of Kaiser with 2105 connections was extended to 2902 connections. The links considered here are all chemical synapses. The formation of gap junctions may require direct contact of cell membrane at certain stage of development, and they are not considered in this study of wiring cost and processing efficiency. The updated network has 276 neurons and 2902 links. The names of the neurons are listed in Table S2 of SI. Here the wiring length 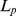 is measured as the Euclidean length between all connected pairs of neurons.

### Methods

### 

#### Objective function and connectivity optimization

We reconstructed network connections for a given total number of connections while fixing the spatial position of the nodes. The reconstructed networks were to minimize an objective function combining the wiring cost and processing efficiency constraints,
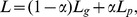



where 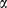 is a parameter to represent the relative weight of the normalized physical wiring length 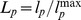 and the normalized graph length 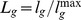. Here 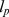 is the total wiring length of the links and 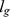 is the sum of the shortest pathlength between all pairs of nodes in the network. 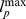 is obtained at 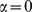 when minimizing 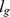 without considering any spatial constraint and 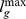 is obtained at 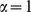 when minimizing 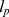 without considering the efficiency constraint.

We applied a simulated annealing optimization algorithm [106] to search for network configurations that minimize the objective function 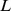. In each step, we set the programme to avoid those choices which made the network disconnected. In the beginning, the simulated annealing approaches were employed to obtain the benchmark networks at 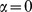 and 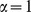 to obtain the physical distance 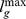 and graph distance 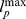, respectively. Using them for normalization, we made the value of the objective function 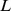 stay within (0, 1].

The simulated annealing optimization algorithm was implemented as follows. We started with a random network and a high temperature 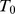, and then decreased temperature as 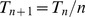. At each temperature, we rewired the network for 1000 steps. At each step, we chose four random nodes to exchange the connections, and accepted the changes with the probability 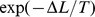. The program was terminated when 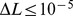. For each 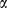, the simulated annealing programme was performed to obtain 50 realizations of the optimized networks from different initial random networks.

We first applied the method to a 1D model with the nodes arranged uniformly on a 1 one-dimensional ring, then to the primate cortico-cortical network of Macaque and the neuronal network of *C. elegans*.

#### 1D model

The nodes were arranged with equal distance on a one-dimensional circle. The links were directed. The wiring length of a connection is the Euclidean distance between two nodes 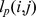 and 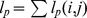.

The optimal solution for 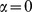 (processing efficiency constraint only) is a network with apparently random connections and a single, global hub; and the optimal solution for 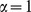 (wiring cost constraint only) is a regular, local graph, while in-between the optimal solutions are networks with local connections and hubs (Fig. 1).

#### Modularity analysis

A modular community is defined as a subnetwork with a higher density of connections relative to the entire network, based on the work by Newman [28]. The modular structure is obtained through optimizing the partitioning of the network into several modules to maximize a quantitative measure of modularity, 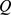, defined as [107]

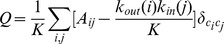



where, 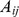 = 1 when there is a directed projection from node 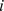 to node 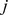, and 0, otherwise. 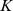 is the total number of links in this network, 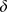 is the Kronecker delta (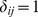 if 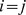, and 0 otherwise), and 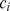 is the index of the community where node 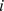 is assigned. We used the modularity-dir.m [108] in (the toolbox of brain connectivity) BCT package for the module partition. Since there could be uncertainty and degeneracy in the algorithm [109], [110], we applied the methods for 50 independent runs for each network considered (real or reconstructed networks). To check the significance of the modularity, we also measured modularity in the corresponding random networks with the same number of nodes and links as in the network examined. In the event, we found that the modular division had no degeneracy in both real networks of Macaque and *C. elegans* and in reconstructed networks of Macaque (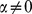), but it had small degeneracy for the reconstructed networks of *C. elegans* (typically 1–5 neurons of mismatch from run to run).

#### Spatial clustering

As seen from two-dimensional projection of the spatial layout and the connectivity of the Macaque cortical network in Fig. 5A, the nodes can be roughly divided into two dense groups which are spatially distant from each other. To perform a quantitative analysis of the spatial clustering, we computed the pair-wise Euclidean distance of the nodes to obtain the distance matrix and applied the hierarchical clustering algorithm in MATLAB (linkage.m and cluster.m) to the distance matrix. With a suitable threshold, we could obtain the same number of clusters as network modules in order to compare them appropriately.

There are various ways to compute distance in agglomerating the hierarchical cluster tree, including *centroid distance, inner squared distance, the shortest distance, the furthest distance, average distance, weighted average distance, and weighted center of mass distance between clusters*. In addition to hierarchical clustering, we also applied the K-means method to minimize the within-cluster sums of point-to-cluster-centroid distances. While the results are deterministic for a given distance measure, they differ slightly for different distance measures. To take this uncertainty into consideration, in this work, we applied the different methods to the real networks of Macaque and *C. elegans*, and compared the clusters from different methods to the modules of the real network obtained by the BCT method (see sections “Modularity analysis” and “Matching between partitions” in *Method*). We found that in both systems, the clusters by inner squared distance has the minimal matching rate with the modules, and the group of mismatched nodes is the largest possible to cover all the mismatching sets in the other methods (centroid distance produced the same result as inner squared distance in Macaque). This means that the set of matching areas resulting from the inner squared distance is most stable when different possible clusterings are considered. Thus we used the clusters from the inner squared distance for further comparison with the modules in reconstructed networks.

The method was applied to the Macaque cortical network to obtain two clusters (Fig. 5) and to the *C. elegans* neuronal network to obtain four clusters (Fig. 6).

For the Macaque cortical network, the comparison of the clustering of two datasets (103 areas and 176 areas) was shown in Fig. S4 and S5 for the dendrograms (A,B) and for the boundaries between the division into two clusters (C). There are only five mismatched areas between the two datasets (areas 4, 6C, 3b, 2/1 and AI according to the dataset of 176 areas) (Fig. S5).

#### Matching between partitions

We compared the partition of spatial clusters with the partition of modules or the partitions of modules in real and reconstructed networks. Suppose the partition A gives 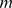 groups 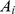 and partition B generates 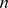 groups 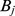 with 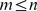. For each partition 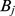, we found the corresponding group in partition A to which the group 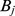 should belong (the one covers the maximal number of nodes in 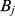). This maximally covered number in 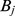 is added to the matched set and the rest of the nodes are counted in the mismatched set. Note that it is possible that two or more groups in B can belong to the same group in A. In this way, we identified all the nodes matching the two partitions. Taking the matching between the clusters and modules of the real *C. elegans* network as an example, there are 3 spatial clusters and 4 modules, and two modules belong to the same cluster at the head.

We also developed a way to judge the significance of matching between partitions by comparing the results to “shuffled modules”. We kept the size of all subgroups 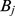 of partition B, but randomly shuffled two elements in different subgroups. Such shuffled data have the same n groups as in the real partition B, but the labels of nodes are randomized. We obtained an ensemble of such shuffled modules and calculated the average mismatching rate with the real partition A. In fact, this average value is independent of the group number 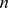 and the size of the groups when the ensemble is large enough. The results are shown as the dashed lines in Fig. 5 F and 6 F.

#### Identifying hubs in networks

There is no unique definition of hubs in networks. In general, the hub nodes should have a degree of about the same order of the network size, much larger than the mean degree of the whole network. Such a picture is usually reasonable when the degree distribution is not very narrow. Taking *C. elegans* for instance, the mean value of in-degree is 10.51, and the standard deviation in the reconstructed networks varies at different 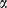, such as 18.2 (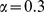), 13.2 (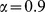), 10.2 (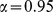) and 10.6 (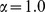). The standard deviation is not small in our work, therefore we heuristically identified hubs in the real and reconstructed networks by the z-score of the degree 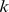 of the node, which is defined as 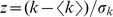, where 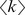 is the average degree of the network and 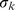 is the standard deviation of the degree distribution. In a previous analysis of hubs in cortical network [20], a node was regarded as a hub when 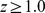. To focus on nodes with the degree significantly larger than the average degree, in our analysis, a node was regarded as a hub when 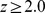. For example, in the reconstructed of *C. elegans* at 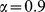, the hubs identified in this way have an average degree of 42, which is reasonably large when compared to the mean degree 10.5 and the standard deviation 10.2 in the network.

Despite the ambiguity in defining hubs in networks, this method provides a pragmatic way to characterize the properties about the changes of the number of nodes with relatively large degrees, the average degree of them and their spatial positions with respect to the parameter 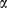, which were quite clear and common across the different networks analyzed.

#### Locality measure 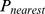 of connections

We measured to what extent the links in the network were connected to spatial nearest neighbors. For each node 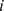, we considered the total number of connections 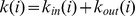. The total wiring length 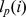 from this node can be computed as the sum of the wiring length of the 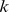 links. 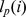 for the node 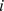 will be minimal, 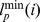, if all the 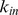 input links and 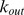 output links are connected to 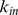 and 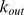 nearest neighbors of the node 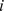, respectively. One can use the ratio 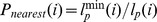 to quantify the locality of the connections of the nodes in the network. All the connections of a node are local if 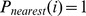. One can obtain a locality measure of the connections of the whole network as the average of 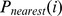, i.e.,
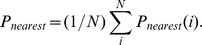




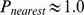
 indicates that most of the connections in the network are to the spatial nearest neighbors. For random connections, 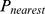 is small. The connections with a distant hub reduce the locality measure of the network.

#### Geographical centrality of the nodes

Under the multiple constraints of wiring cost and processing efficiency, the centrality of a node in the spatial layout of the network, called *geographical centrality* here, may be closely related to its connectivity. To quantify the geographical centrality, we computed the total Euclidean distance 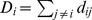 from a node 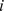 to all the other nodes in the network, and ranked the nodes according to this distance 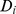. The node with the smallest total distance has the highest geographical centrality.

We can also measure the geographical centrality with respect to a subset of nodes, for example, within one of the spatial clusters of nodes.

#### Neighborhood density of nodes in space

Unlike the 1D model, the spatial distribution of the nodes in the original Macaque cortical network is not uniform due to varying sizes and shapes of the cortical areas. The neurons in *C. elegans* are also distributed highly non-uniformly in space. To quantify the spatial heterogeneity, we evaluated the density of nodes in the neighborhood of a given node, called the neighborhood density 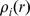, i.e.,
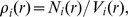



where 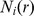 represents the number of nodes lying within the sphere (for Macaque) or circle (for *C. elegant*) of radius 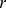, centered at the given node 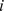, and 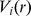 is the volume of the sphere (area of the circle).

The radius 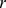 is referenced to the maximal distance 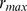 between two nodes in the network.

#### Comparing the connectivity of reconstructed and real networks and computing the recovery rate

The most direct way to compare the connectivity of the reconstructed network with the original network is to compare the adjacency matrices using measures such as the Hamming distance. One can measure the number of overlapping entries 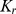 of the two adjacency matrices and obtain the recovery rate as 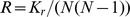 (without considering the diagonal entries). However, this result could be biased when the number of 1's (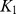) and the number of 0's (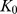) are strongly asymmetrical in the network. This is the case for both the Macaque cortical network (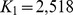 and 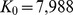) and *C. elegans* neuronal network (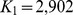 and 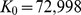). We followed the method proposed in [27] to compute the recovery rate as 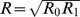 where 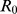 and 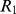 are the recovery rate for 1's and 0's, respectively. In particular, 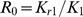 and 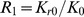, where 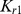 and 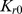 are the number of overlapping entries with value 1 and 0, respectively.

The same method can be applied to obtain the recovery rate for subnetworks by considering the corresponding sub-matrices, for example, the connections within and between the spatial clusters or the modules (Fig. 10C,D). It can also be extended to the connections between nodes separated by a distance with certain range ((Fig. 10E,F).

#### Testing whether degrees follow power-law distribution

We used the Kolmogorov-Smirnov statistic and maximum-likelihood fitting methods (MLEs) [111], to test whether the degree distribution in real or reconstructed networks can be fitted by a power-law function. First, the following power-law model
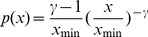



is fitted to the data to estimate the parameters 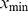 and 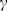. Then an ensemble of surrogate data (with the same number of data points as in the degree distribution) is generated from the above power-law function with the estimated 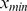 and 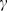. The distance 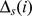 between the 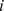
th surrogate data set and the model function can be obtained and compared to the distance 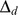 between the degree distribution and the model. A 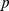 value is obtained as the fraction of surrogate data sets in the ensemble with 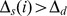. Thus if 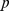 is large (close to 1), then the difference between the degree distribution and the power-law model can mainly be attributed to the statistical fluctuation. But if 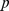 is small (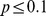), then the power-law can be ruled out for this degree distribution.

#### Significance test of correlation

We studied several types of correlations between two data sets of small sizes, e.g., correlation between degrees and density in the reconstructed and real networks. To evaluate the significance of the correlation between two finite-size data sets, we used the method of surrogate data. We randomly reshuffled the order of one of the data sets and computed the correlation values for 100 realizations of the random reshuffling. From the distribution of the correlation of such surrogate data one can obtain the standard deviation 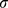. If the correlation between the two original data sets is outside 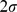 of this distribution, it has about 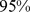 of significance that the real correlation is not a coincidence due to finite size of the data sets.

## Supporting Information

Figure S1 **The adjacency matrix of the Macaque cortical networks with finer division of the motor areas.** The links in the original data set of Kaiser are shown as gray, and the links from or to new, finer motor areas are shown by blue.
(EPS)

Figure S2
**Adjacent matrices of the networks with various connection probabilities in 1D model at** 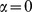 **.** A single hub appears for sparse networks (A, 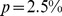) and (B, 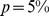), but not in dense network (C, 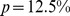).(EPS)

Figure S3
**Input and output degrees in reconstructed and real networks.** (A) Reconstructed network of the Macaque cortical network, with 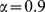. (B) Real network of the Macaque cortical network. The correlation between the input and output degrees is 0.589 which is larger than 95% significance level at 0.237 in the corresponding randomly reshuffled data (see *Materials and Methods*). (C) Reconstructed network of the *C. elegans* neuronal network, with 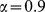. (D) Real network of the *C. elegans* neuronal network. The correlation between the input and output degrees is 0.717, larger than 95% significance level at 0.158 in the corresponding shuffled data.(EPS)

Figure S4
**Comparison of the spatial clustering in two different databases of Macaque cortical network.** (A) and (B) display the clustering dendrogram for datasets with 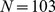 and 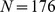 areas, respectively. In both datasets, the nodes can be divided into two pronounced clusters.(EPS)

Figure S5 **The spatial layout of the areas of Macaque cortex.** It shows that the spatial layout of the areas(indicated by lines for 103 areas), with reference to parcellation of 176 areas (color). The boundaries between the two clusters are shown by bold lines (blue for for dataset of 103 areas and red line for 176 areas). These two boundaries are close to each other, indicating that spatial clustering is a robust property in the spatial layout of the cortical areas.
(TIF)

Figure S6
**Modularity** 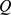 **of reconstructed networks as a function of** 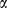 **, compared to** 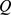 **of the real network.** (A) Macaque cortical network. (B) *C. elegans* neuronal network. The corresponding random networks with the same number of nodes and connections have 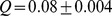 (Macaque) and 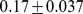 (*C. elegans*). The error-bars are from 50 realizations of the reconstructed networks.(EPS)

Figure S7
**The ranking of a few nodes with the largest total degrees in real Macaque cortical networks in terms of neighborhood density and geographical centrality.** (A) 7 nodes (cortical ares) with the largest total degree. (B) 20% of network nodes having top neighborhood density in a range of radius 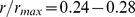. (C) and (D) 40% of nodes from the red and blue clusters having top geographical centrality from the respective cluster (gray bars). The length of the bar indicates the corresponding values. The dark bars in (C) and (D) highlight the cortical areas listed in (A). The nodes with the largest total degrees are among the top list of neighborhood density as well as geographical centrality, which is a strong signature of wiring cost constraint.(EPS)

Figure S8
**Closeness between reconstructed and real hubs in** ***C. elegans*** **.** The bars show the total distance of each of the 166 neurons in the head to the 4 real hubs (AVAL/R and AVBL/R, red and blue bullets), sorted from small to large values. Green triangles indicated the hubs in the reconstructed networks for 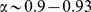.(EPS)

Figure S9
**Degree distribution in the original and reconstructed networks.** (A) shows the outdegree distribution for Macaque cortical network. (B) shows the outdegree distribution for *C. elegans* neuronal network. The degree distribution in reconstructed networks does not differ significantly for different 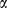 values, since the degrees are mainly determined by the neighborhood density of the node. In the insets, the dashed line with different color represents the fitting power-law distribution from the corresponding degree distribution with the same color (including distributions not obeying power-law). Inset of A: 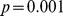 (real network), 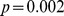 (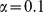), 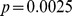 (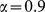). Inset of B: 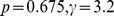 (real network), 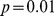 (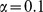), 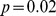 (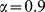).(EPS)

Figure S10
**Degrading effect on processing efficiency when removing the node with the largest degree in reconstructed networks.** Shown is the ratio between 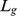 after and before the removal of the node. (A) Macaque cortical network. (B) *C. elegans* neuronal network. The error-bars are from 50 realizations of the reconstructed networks.(EPS)

Table S1 **The list of the labels of 103 cortical areas in Macaque is provided on-line.**
(XLS)

Table S2 **The list of the names of 276 neurons in** ***C. elegans*** **is provided on-line.**
(XLS)
