# Supplementary figures and images for: Trade-off between Multiple Constraints Enables Simultaneous Formation of Modules and Hubs in Neural Systems
Source: PLoS Comput Biol. 2013 Mar 7;9(3):e1002937. doi: 10.1371/journal.pcbi.1002937 (PMC3591279; doi:10.1371/journal.pcbi.1002937)

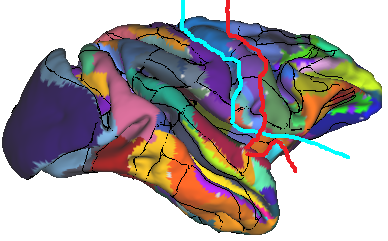

Supplement: Figure S5 — The spatial layout of the areas of Macaque cortex. It shows that the spatial layout of the areas(indicated by lines for 103 areas), with reference to parcellation of 176 areas (color). The boundaries between the two clusters are shown by bold lines (blue for for dataset of 103 areas and red line for 176 areas). These two boundaries are close to each other, indicating that spatial clustering is a robust property in the spatial layout of the cortical areas. (TIF) [file pcbi.1002937.s005.tif]
